# Diagnostic accuracy of *Mycobacterium tuberculosis*-specific triple-color FluoroSpot assay in differentiating tuberculosis infection status in febrile patients with suspected tuberculosis

**DOI:** 10.3389/fimmu.2024.1462222

**Published:** 2025-01-08

**Authors:** Lifan Zhang, Yuanchun Li, Xiaoqing Zou, Huimin Ma, Mengqiu Gao, Qiping Ge, Yueqiu Zhang, Zhengrong Yang, Xinuo Song, Qiwen Yang, Xiaoqing Liu

**Affiliations:** ^1^ Division of Infectious Diseases, Department of Internal Medicine, State Key Laboratory of Complex Severe and Rare Disease, Peking Union Medical College Hospital, Chinese Academy of Medical Sciences and Peking Union Medical College, Beijing, China; ^2^ Clinical Epidemiology Unit, Peking Union Medical College, International Clinical Epidemiology Network, Beijing, China; ^3^ Center for Tuberculosis Research, Chinese Academy of Medical Sciences and Peking Union Medical College, Beijing, China; ^4^ Department of Scientific Research, Xiangya Hospital, Central South University, Changsha, China; ^5^ Department of Tuberculosis, Beijing Chest Hospital, Capital Medical University/Beijing Tuberculosis and Thoracic Tumor Research Institute, Beijing, China; ^6^ Department of Clinical Laboratory, Peking Union Medical College Hospital, Chinese Academy of Medical Sciences, Beijing, China

**Keywords:** FluoroSpot, triple-color, T-SPOT.TB, active tuberculosis, latent tuberculosis infection

## Abstract

**Objective:**

This study aims to evaluate the diagnostic accuracy of a *Mycobacterium tuberculosis* (MTB)-specific triple-color FluoroSpot assay (IFN-γ/IL-2/TNF-α) in the differentiation of tuberculosis (TB) infection status in febrile patients.

**Method:**

Febrile patients with suspected active TB (ATB) were consecutively enrolled. The frequencies and proportions of MTB-specific T cells secreting IFN-γ, IL-2, and TNF-α were detected at the single-cell level by triple-color FluoroSpot assay. The diagnostic index was fitted with a binary logistic regression model, and the diagnostic accuracy was evaluated according to the receiver operating characteristic (ROC) curve. The sensitivity, specificity, predictive values (PV), and likelihood ratios (LR) were calculated.

**Result:**

A total of 210 febrile patients were enrolled, 53 patients were diagnosed with ATB (28 pathogen-confirmed vs. 25 clinically diagnosed) and 157 patients were non-ATB (84 with latent tuberculosis infection (LTBI) vs. 73 uninfected with MTB). Additionally, 30 pathogen-confirmed ATB patients were assembled. When diagnosing ATB, the area under the ROC curve (AUROC) of the MTB-specific triple-color FluoroSpot assay was significantly better than that of T-SPOT.TB (0.882 vs. 0.811, *p* = 0.017). With the fitted diagnostic index at a cutoff value of 0.378, the sensitivity, specificity, LR+, and LR- were 74.7%, 93.0%, 10.66, and 0.27, respectively. When differentiating ATB from LTBI, the AUROC of the FluoroSpot assay and T-SPOT.TB was 0.878 and 0.692, respectively (*p* < 0.001). With a diagnostic index of 0.413, the sensitivity, specificity, LR+, and LR were 77.1%, 85.7%, 5.40, and 0.27, respectively.

**Conclusion:**

The MTB-specific triple-color FluoroSpot (IFN-γ/IL-2/TNF-α) might be helpful for the differentiation of TB infection status in febrile patients.

## 1 Introduction

Tuberculosis (TB) is a chronic infectious disease caused by *Mycobacterium tuberculosis* (MTB), which is mainly transmitted by the respiratory tract and can invade multiple human organs, seriously endangering human health. The World Health Organization (WHO) estimated that there were nearly 10.6 million new cases of active tuberculosis (ATB) worldwide, with about 1.6 million deaths, second only to coronavirus disease 2019 (COVID-19) and ranking second in the cause of death of a single infectious disease. There were 780,000 newly diagnosed TB patients and 30,000 deaths in 2021 in China, whose burden of TB ranks third in the world (1). Among people infected with MTB, 90% do not develop ATB directly. They instead go into a latent tuberculosis infection (LTBI) status without any clinical symptom (2). This special infectious status accounted for almost 20% of the population and was estimated to be more than 300 million people in China (3).

Early diagnosis of ATB is very important to improve the cure rate and patients’ prognosis and reduce the fatality rate and the risk of transmission. However, WHO estimated that only about 42% of pulmonary TB patients were diagnosed clinically, and Pang Y’s study showed that in China more than 87% of extrapulmonary TB patients were diagnosed based on the epidemiological history, clinical symptoms, imaging findings, laboratory examination including immunological assays, and response to empirical anti-TB treatment (4, 5). Fever is one of the most common clinical manifestations of ATB, and ATB is also the key point in the clinical differential diagnosis in febrile patients. It is challenging to distinguish ATB from non-ATB in febrile patients without etiological evidence.

Interferon-gamma release assay (IGRA), a method to diagnose TB infection by detecting the secretion of interferon-gamma (IFN-γ) stimulated with MTB-specific antigen, is unable to distinguish ATB and LTBI (6). In addition to IFN-γ, interleukin 2 (IL-2) and tumor necrosis factor alpha (TNF-α) also play essential roles in the cellular immune response of MTB infection (7). It was found that the secretion of IL-2, IFN-γ, and TNF-α varied in different stages of MTB infection (8–12), which suggested that a comprehensive analysis of multiple MTB-specific cytokines’ secretion is expected to identify the TB infection status. Our previous studies showed that the simultaneous detection of IFN-γ and IL-2 or IFN-γ and TNF-α secretion at the single-cell level by dual-color FluoroSpot assay helped to distinguish ATB from non-ATB (13–15). A prospective study in Korea found that the sensitivity and specificity of the IFN-γ/TNF-α dual release assay by FluoroSpot were reported as 84% and 94% to diagnose active TB, respectively (16). However, there is no published study on the simultaneous detection of MTB-specific IFN-γ, IL-2, and TNF-α cytokines at the single-cell level by triple-color FluoroSpot assay.

In this study, a MTB-specific triple-color FluoroSpot assay was established to detect IFN-γ, IL-2, and TNF-α simultaneously at the single-cell level and preliminarily evaluate its diagnostic accuracy in the differentiation of TB infection status in febrile patients.

## 2 Methods

### 2.1 Study design and participants

A cross-sectional study was designed. We consecutively included febrile patients with suspected ATB from the outpatient and inpatient departments in Peking Union Medical College Hospital from April 2020 to August 2021. We additionally included ATB patients who were confirmed by etiology in Beijing Chest Hospital during the same period. The inclusion criteria for febrile patients included age ≥18 years old and with fever symptoms (oral temperature exceeds 37.3°C or the rectal temperature exceeds 37.8°C) (17, 18), and the exclusion criteria included HIV infection, pregnancy or lactation, and hematologic malignancies. The diagnostic classification of TB infection status was predefined as presented in **Table 1**. Given the prospective nature of this study, all participants underwent T-SPOT.TB and triple-color FluoroSpot (IL-2/TNF-α/IFN-γ) assays simultaneously using freshly collected peripheral blood immediately after enrollment.

**Table 1:** Predefined diagnostic criteria of TB infection.

Categories	Criteria
ATB	
Pathogen-confirmed ATB	① *MTB* culture or/and acid-fast staining or/and Xpert *MTB*/RIF or/and *MTB* nucleic acid positive and ② Clinical manifestations of ATB (fever, cough, night sweats or weight loss, etc.) and ③ Laboratory examination and imaging findings suggesting ATB and ④ Untreated or had received ≤2 weeks of anti-TB treatment
Clinically diagnosed ATB	① No radiological or etiological evidence of ATB and ② Clinical manifestations of ATB (fever, cough, night sweats or weight loss, etc.) and ③ Laboratory examination and imaging findings suggesting ATB and ④ Empirical anti-TB treatment is effective
Non-ATB	
LTBI	① No radiological or etiological evidence of ATB and ② T-SPOT.TB test was positive and ③ Other diseases were confirmed and responded to treatment
Not infected with *MTB*	① No radiological or etiological evidence of ATB and ② T-SPOT.TB test was negative and ③ Other diseases were confirmed and responded to treatment

### 2.2 Reference standard

The reference standard for ATB included pathogen-confirmed and clinically diagnosed status, respectively. ATB patients who were clinically diagnosed were independently diagnosed by two senior specialists based on the patients’ clinical manifestations, laboratory examination, imaging findings, and response to empirical anti-TB therapy (19. A superior specialist would join to make the final diagnosis when a disagreement occurred. All physicians involved were only aware of the T-SPOT.TB results and were not informed of the MTB-specific triple-color FluoroSpot (IFN-γ/IL-2/TNF-α) test results.

### 2.3 The index test

#### 2.3.1 Collection of samples

A total of 8 mL of peripheral venous blood was collected from the participants and evenly distributed into two heparin lithium-anticoagulant tubes. Triple-color FluoroSpot (IFN-γ/IL-2/TNF-α) assay and T-SPOT.TB were tested simultaneously. Peripheral blood mononuclear cells (PBMCs) were isolated within 6 h after sample collection, and a cell suspension of 2.5 × 10^6^ cells/mL was prepared with AIM-V (GIBCO™ AIM-V Medium Liquid, Invitrogen, USA).

#### 2.3.2 T-SPOT.TB

T-SPOT.TB test was performed using a commercially available kit (Oxford Immunotec Ltd., Oxford, UK) according to the manufacturer’s instructions. Spot-forming cells (SFCs) were counted with an automated ELISpot reader (AID-iSpot, Germany).

#### 2.3.3 Triple-color FluoroSpot (IL-2/TNF-α/IFN-γ) assay

**Figure 1:**
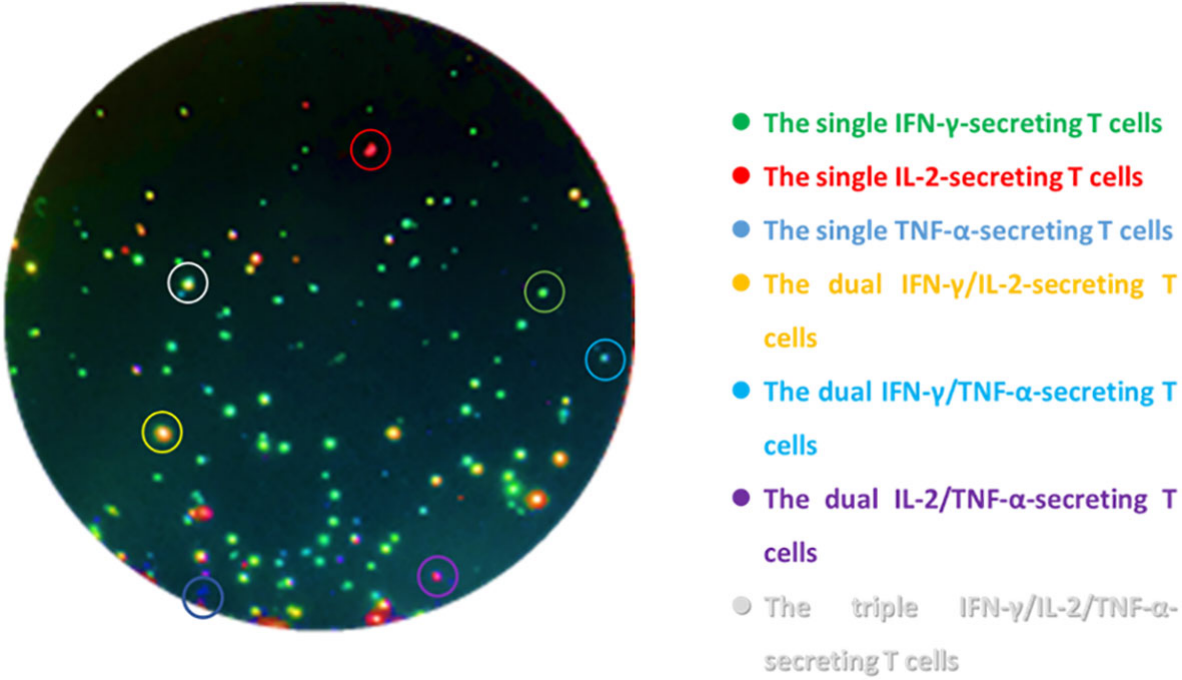
Schematic diagram of triple-color FluoroSpot (IFN-γ/IL-2/TNF-α) detecting cytokine-secreting specific T cells after stimulation with MTB-specific antigens. The green spots are the single IFN-γ-secreting T cells, the red spots are the single IL-2-secreting T cells, the blue spots are the single TNF-α-secreting T cells, the yellow spots are the dual IFN-γ/IL-2-secreting T cells, the cyan spots are the dual IFN-γ/TNF-α-secreting T cells, the purple spots are the dual IL-2/TNF-α-secreting T cells, and the white spots are the triple IFN-γ/IL-2/TNF-α-secreting T cells.

Triple-color FluoroSpot (IL-2/TNF-α/IFN-γ) (Mabtech AB, Sweden) assays were performed according to the manufacturer. The 96-well plates which were pre-coated with monoclonal antibodies against IFN-γ, IL-2, and TNF-α were seeded with 2.5 × 10^5^ PBMCs and anti-CD28 (0.1 µg/mL, Mabtech AB, Sweden) and contained AIM-V (GIBCO™ AIM-V Medium Liquid, Invitrogen, USA) as nil control, PHA (5 μg/mL) as positive control, and peptides of ESAT-6 (10 μg/mL) and CFP-10 (10 μg/mL) as MTB-specific antigens (self-synthesized, Beijing Zhongke Yaguang Biotechnology Co., Ltd.). The plates were incubated for 16–20 h at 37°C in 5% carbon dioxide and then washed with PBS buffer. A total of 100 µL of the biotinylated and/or tag-labeled detection antibodies of anti-IFN-γ-BAM, anti-IL-2-MT8G10, and anti-TNF-α-WASP (the dilutions were 1:200, 1:500, and 1:200, respectively) was added to each well; then, 2 h of incubation at room temperature under dark conditions was performed. Next, the plates were washed with PBS buffer again; then, 100 uL of anti-bam-490, anti-550, and anti-wasp-640 mixture (the dilutions were 1:200) was added, and 1 h of incubation in the dark was needed. Finally, the fluorescent enhancer was loaded and incubated for 15 min. ELISpot reader (AID-iSpot, Germany) was used to read the frequencies of T cells: ① the total IFN-γ-secreting T cell, ② the total IL-2-secreting T cells, ③ the total TFN-α-secreting T cells, ④ the dual IFN-γ/IL-2-secreting T cells, ⑤ the dual IFN-γ/TNF-α-secreting T cells, ⑥ the dual IL-2/TNF-α-secreting T cells, and ⑦ the triple IFN-γ/IL-2/TNF-α-secreting T cells. The MTB-specific T cells secreting different cytokine profiles appeared as spots of different colors of fluorescence (**Figure 1**), the frequencies and proportions of which were calculated from the abovementioned data (**Supplementary Figure S1**). The laboratory staff who conducted the assays were all blinded to the patients’ clinical data.

### 2.4 Statistical analysis

It was assumed that *MTB-*specific triple-color FluoroSpot (IFN-γ/IL-2/TNF-α) has 85% sensitivity and 90% specificity in diagnosing ATB from uninfected patients and 80% sensitivity and 75% specificity in differentiating ATB and LTBI (13, 14, 20). A value of *P <*0.05 was considered significant, and the allowable error was 0.1. By calculation, the minimum sample sizes of the ATB group, LTBI group, and uninfected group should contain 62 cases, 73 cases, and 35 cases, respectively. Since most patients had an unclear diagnosis before undergoing the index test, we included as many patients as possible to ensure that the actual sample size obtained in each group met the requirements of the calculated sample size.

Continuous variables with normal distribution were presented as mean ± standard deviation (SD), while variables with non-normal distribution were denoted as median and interquartile range (IQR). Enumeration data were presented by percentages and 95% confidence intervals (CIs).The correlation between the frequencies of total IFN-γ-secreting T cells detected by triple-color FluoroSpot assay and T-SPOT.TB was evaluated by using the Spearman correlation coefficient. The probability of ATB for each participant as “diagnostic index” was fitted with a binary logistic regression model (forward LR, entry 0.05, removal 0.10) by using the frequencies and proportions of MTB-specific T cells secreting different cytokine profiles as independent variables, and the diagnostic accuracy of MTB-specific triple-color FluoroSpot (IFN-γ/IL-2/TNF-α) was evaluated according to receiver operating characteristic (ROC) curve analysis by using the diagnostic index. The optimal cutoff value is the value that maximizes the Youden index (sensitivity + specificity - 1). The sensitivity, specificity, PV, and LR values were calculated at the optimal cutoff. Statistical analyses were performed using SPSS 26 (IBM Crop, SPSS Inc., Chicago, IL, USA) and MedCalc (version 11, MedCalc Software bvba, Mariakerke, Belgium). The scatter plot of triple-color FluoroSpot (IFN-γ/IL-2/TNF-α) and T-SPOT.TB was performed using R (version 4.0.5, 2021 The R Foundation for Statistic Computing). *P <*0.05 (two-sided) was considered statistically significant.

### 2.5 Ethical approval

The study was conducted in accordance with the Declaration of Helsinki and was approved by the Ethics Committee of Peking Union Medical College Hospital (no: JS-2350). Informed written consent was obtained from all patients prior to their enrollment in this study.

## 3 Result

### 3.1 Demographic and clinical characteristics of participants

**Figure 2:**
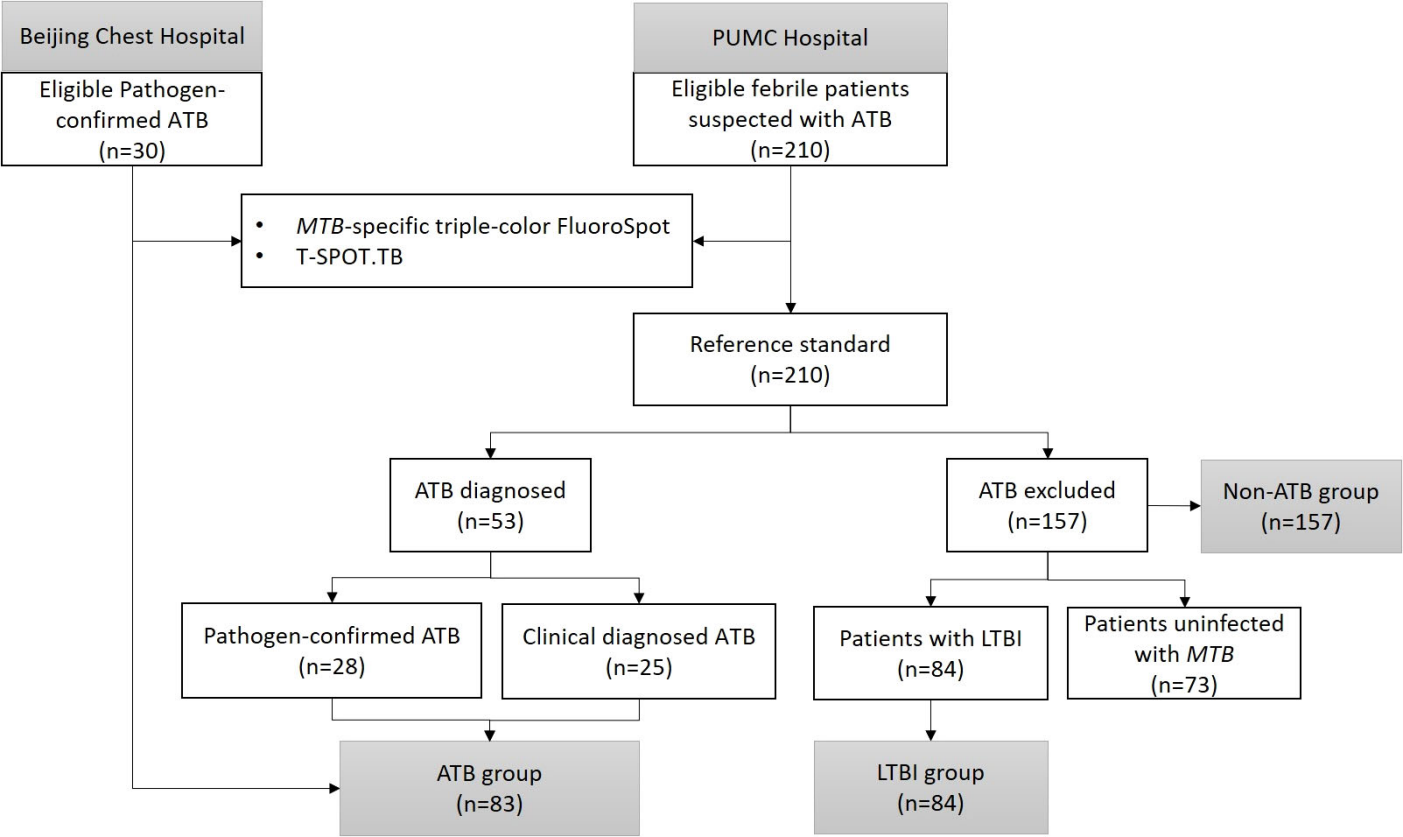
Flow diagram.

A total of 83 febrile patients with ATB (58 etiologically confirmed and 25 clinically confirmed) and 157 febrile patients non-ATB (84 patients with LTBI and 73 patients uninfected with MTB) were included in the study. The process of participant inclusion is shown in **Figure 2**. Among patients excluded from ATB, 44 (28.0%) were diagnosed with other infectious diseases, 74 (47.1%) with rheumatic diseases, 17 (10.8%) with solid tumors, and 22 (14.0%) with other diseases (**Table 2**). The basic characteristics of the participants are shown in **Table 2**.

**Table 2:** Demographic and clinical characteristics of the participants.

	ATB (*N* = 83)	LTBI (*N* = 84)	Uninfected *MTB* (*N* = 73)
Age (median, IQR)	55 (39, 63)	56 (46, 64)	50 (30, 62)
Male (*n*, %)	48 (59.0)	51 (59.5)	25 (34.2)
Laboratory examination			
WBC*10^9^/L (median, IQR)	6.03 (4.87, 8.08)	7.13 (5.31, 9.75)	6.78 (5.12, 9.30)
NE#*10^9^/L (median, IQR)	4.55 (3.28, 6.06)	4.54 (3.22, 6.52)	4.86 (2.93, 7.38)
LY#*10^9^/L (median, IQR)	1.24 (0.84, 1.77)	1.69 (1.21, 2.17)	1.43 (0.94, 1.90)
Hb*g/L (mean ± SD)	118 ± 23	122 ± 25	110 ± 26
PLT*10^9^/L (median, IQR)	307 (229, 369)	228 (185, 296)	239 (180, 334)
ALT*U/L (median, IQR)	15 (11, 26)	19 (13, 28)	17 (11, 38)
Tbil*μmol/L (median, IQR)	9.4 (7.0, 12.0)	9.9 (7.4, 13.7)	8.3 (6.5, 12.5)
Alb*g/L (median, IQR)	36 (31, 39)	39 (33, 42)	35 (31, 40)
Cr*μmol/L (median, IQR)	61.2 (53.0, 74.7)	70.0 (60.0, 86.0)	62.0 (50.0, 78.0)
hsCRP*mg/L (median, IQR)	29.99 (5.06, 62.30)	4.02 (0.95, 36.06)	10.26 (2.54, 56.90)
Medications (recent 3 months)			
GCs (*n*, %)	10 (12.0)	29 (34.5)	28 (38.4)
Immunosuppressant (*n*, %)	7 (8.4)	23 (27.4)	22 (30.1)
Biological agent (*n*, %)	1 (1.2)	1 (1.2)	3 (4.1)
Clinical diagnoses			
Pulmonary TB	50 (60.2)	0	0
Extra-pulmonary TB	29 (34.9)	0	0
TB with uncertain site	4 (4.8)	0	0
Infectious diseases	0	24 (28.6)	20 (27.4)
Autoimmune diseases	0	41 (48.8)	33 (45.2)
Solid tumor	0	10 (11.9)	7 (9.6)
Others			
Physiological fever	0	2 (2.4)	6 (8.2)
Systemic autoinflammatory diseases	0	1 (1.2)	3 (4.1)
Kidney disease	0	3 (3.6)	1 (1.4)
Lung disease	0	1 (1.2)	2 (2.7)
Sarcoidosis	0	0	1 (1.4)
Metabolic myopathies	0	0	1 (1.4)
Lymphoproliferative disease	0	1 (1.2)	0

WBC, white blood cell; NE#, neutrophil; LY#, lymphocyte; Hb, hemoglobin; PLT, platelet count; ALT, alanine aminotransferase; Tbil, total bilirubin; Alb, albumin; Cr, creatinine; hsCRP, high-sensitivity C-reactive protein; GCs, glucocorticoids.

### 3.2 Correlation of MTB-specific IFN-γ response between the triple-color FluoroSpot (IFN-γ/IL-2/TNF-α) assay and T-SPOT.TB

**Figure 3:**
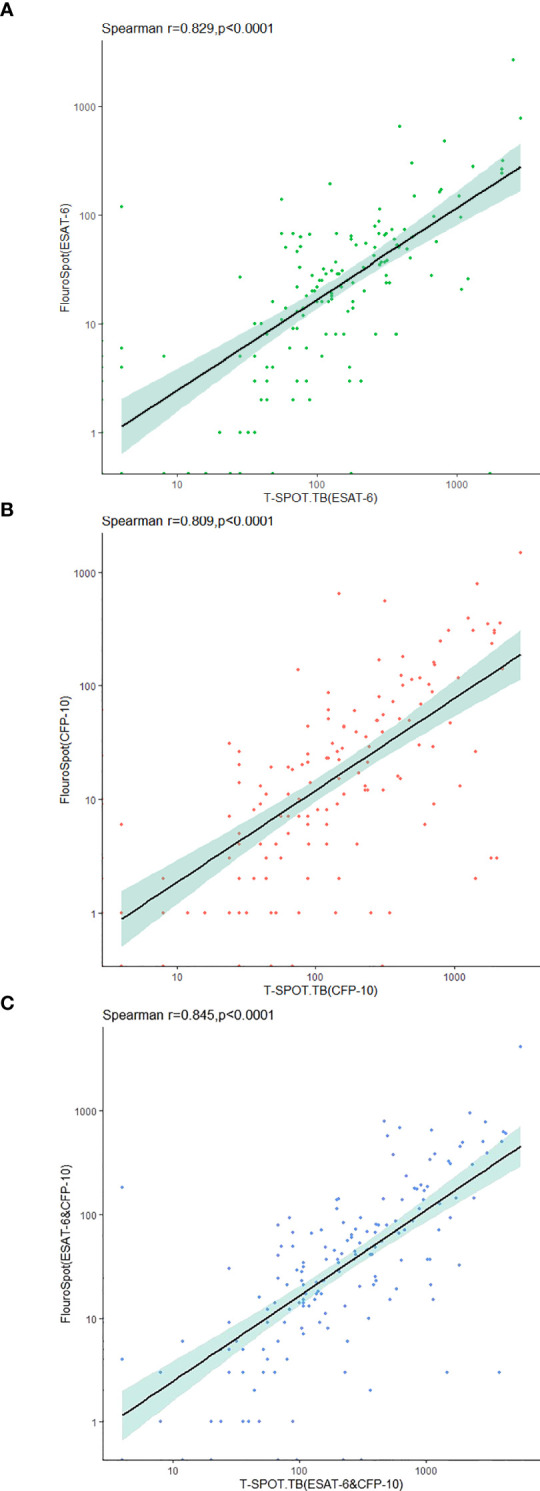
Correlation between triple-color FluoroSpot (IFN-γ/IL-2/TNF-α) and T-SPOT.TB in the detection of IFN-γ-secreting specific T cells stimulated with MTB-specific antigen. **(A)** Frequency of specific T cells secreting IFN-γ after stimulation with ESAT-6. **(B)** Frequency of specific T cells secreting IFN-γ after stimulation with CFP-10. **(C)** Sum of IFN-γ-secreting specific T cell frequencies after stimulation with ESAT-6 and CFP-10.

The frequencies of the total IFN-γ-secreting T cells after being stimulated with ESAT-6 and CFP-10 were detected by triple-color FluoroSpot (IFN-γ/IL-2/TNF-α) assay and T-SPOT.TB. The results showed a strong and significant correlation between the two assays for IFN-γ secretion (**Figure 3**).

### 3.3 Accuracy of MTB-specific triple-color FluoroSpot (IFN-γ/IL-2/TNF-α) and T-SPOT.TB in the diagnosis of ATB

**Figure 4:**
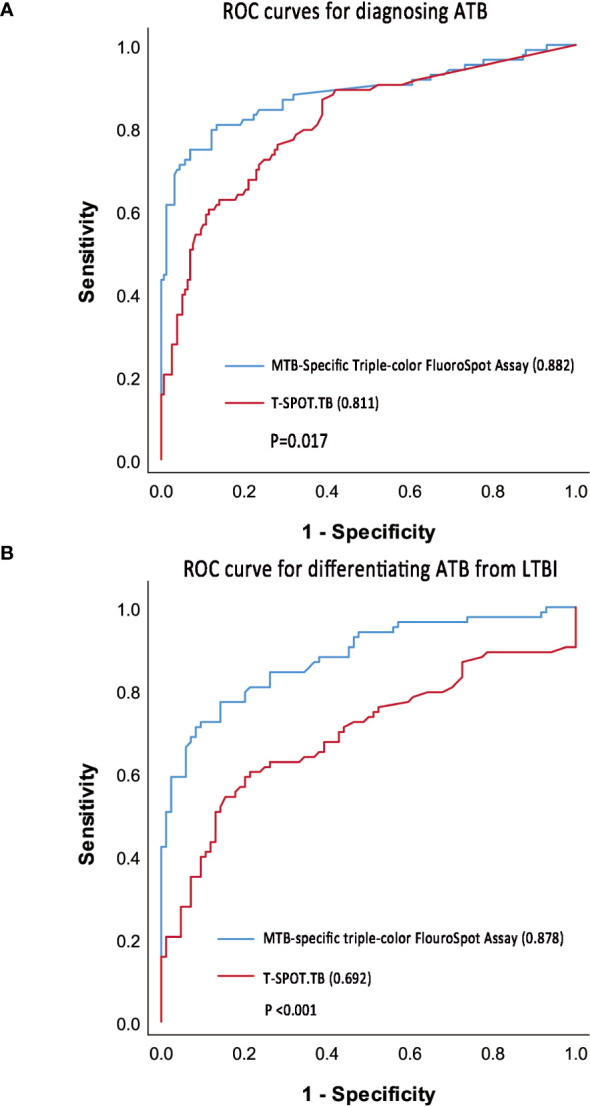
ROC curve of triple-color FluoroSpot (IFN-γ/IL-2/TNF-α) and T-SPOT.TB. **(A)** ROC curves to diagnosing ATB. **(B)** ROC curve to differentiate ATB from LTBI.

In the ATB and non-ATB group, a binary logistic regression model was established by using the frequencies and proportions of T cells secreting different cytokine profiles under ESAT-6 and CFP-10 stimulation as independent variables. Taking the frequencies of IFN-γ^+^IL-2^-^TNF-α^+^-, IFN-γ^+^IL-2^-^TNF-α^–^, and IFN-γ^-^IL-2^+^TNF-α^–^specific T cells and the proportions of IFN-γ^+^IL-2^+^TNF-α^–^specific T cells as the fitting diagnostic index, the AUROC of MTB-specific triple-color FluoroSpot (IFN-γ/IL-2/TNF-α) to diagnose ATB was 0.882 (95%CI: 0.829–0.935), which was significantly better than that in T-SPOT.TB (AUROC = 0.811, 95%CI: 0.753–0.870, *P* =0.017) (**Figure 4A**). The logistic regression results are shown in **Supplementary Table S1**.

Taking 0.378 as the optimal cutoff value of the diagnostic index, the sensitivity and specificity of the MTB-specific triple-color FluoroSpot (IFN-γ/IL-2/TNF-α) assay to diagnose ATB were 74.7% (95%CI: 0.640-0.836) and 93.0% (95%CI: 0.878–0.965), respectively. Taking 302 SFCs/10^6^ PBMCs as the optimal cutoff value, the sensitivity and specificity of T-SPOT.TB to diagnose ATB were 60.2% (95%CI: 0.489–0.708) and 88.5% (95%CI: 0.825–0.931), respectively (**Table 3**). Furthermore, the difference in sensitivity between triple-color FluoroSpot (IFN-γ/IL-2/TNF-α) and T-SPOT.TB was statistically significant (*P* = 0.004), while that in specificity was not significant (*P* = 0.118).

**Table 3:** Diagnostic accuracy of *MTB*-specific triple-color FluoroSpot (IFN-γ/IL-2/TNF-α) and T-SPOT.TB.

Test	Sensitivity (%)	Specificity (%)	PLR	NLR
	(95%CI)	(95%CI)	(95%CI)	(95%CI)
*MTB*-specific triple-color FluoroSpot^a^				
Diagnosis of ATB	74.7 (64.0–83.6)	93.0 (87.8–96.5)	10.66 (5.95–19.11)	0.27 (0.19–0.39)
Pathogen-confirmed ATB	81.0 (68.6–90.1)	93.0 (87.8–96.5)	11.57 (6.45–20.73)	0.20 (0.12–0.35)
Clinical diagnosed ATB	60.0 (38.7–78.9)	93.0 (87.8–96.5)	8.56 (4.45–16.46)	0.43 (0.27–0.70)
Differential diagnosis of ATB and LTBI	77.1 (66.6–85.6)	85.7 (76.4–92.4)	5.40 (3.16–9.23)	0.27 (0.18–0.40)
Pathogen-confirmed ATB	81.0 (68.6–90.1)	85.7 (76.4–92.4)	5.67 (3.31–9.72)	0.22 (0.13–0.38)
Clinical diagnosed ATB	68.0 (46.5–85.1)	85.7 (76.4–92.4)	4.76 (2.64–8.58)	0.37 (0.21–0.67)
T-SPOT.TB^b^				
Diagnosis of ATB	60.2 (48.9–70.8)	88.5 (82.5–93.1)	5.25 (3.29–8.39)	0.45 (0.34–0.59)
Pathogen-confirmed ATB	65.5 (51.9–77.5)	88.5 (82.5–93.1)	5.71 (3.56–9.17)	0.39 (0.27–0.56)
Clinical diagnosed ATB	48.0 (27.8–68.7)	88.5 (82.5–93.1)	4.19 (2.31–7.60)	0.59 (0.40–0.86)
Differential diagnosis of ATB and LTBI	60.2 (48.9–70.8)	78.6 (68.3–86.8)	2.81 (1.80–4.39)	0.51 (0.38–0.67)
Pathogen-confirmed ATB	65.5 (51.9–77.5)	78.6 (68.3–86.8)	3.06 (1.95–4.80)	0.44 (0.30–0.64)
Clinical diagnosed ATB	48.0 (27.8–68.7)	78.6 (68.3–86.8)	2.24 (1.26–3.99)	0.66 (0.45–0.98)

The optimal cutoff value was defined as the value that maximizes the Youden index (sensitivity + specificity - 1).

a When diagnosing ATB, a diagnostic index more than 0.378 was defined as positive; when differentiating ATB from LTBI, a diagnostic index more than 0.413 was defined as positive.

b The frequency of spots being more than 302 SFCs/10^6^ PBMCs was defined as positive.

### 3.4 Accuracy of MTB-specific triple-color FluoroSpot (IFN-γ/IL-2/TNF-α) and T-SPOT.TB in distinguishing ATB from LTBI

In the ATB and LTBI group, a binary logistic regression model was established with the frequencies and proportions of T cells secreting different cytokine profiles under ESAT-6 and CFP-10 stimulation as independent variables. When the frequencies of IFN-γ^+^IL-2^-^TNF-α^+^-, IFN-γ^+^IL-2^-^TNF-α^–^, and IFN-γ^-^IL-2^+^TNF-α^–^specific T cells and the proportions of IFN-γ^+^IL-2^+^TNF-α^–^specific T cells were included in fitting the diagnostic index, the AUROC of MTB-specific triple-color FluoroSpot (IFN-γ/IL-2/TNF-α) assay in differentiating ATB from LTBI was 0.878 (95%CI 0.826–0.931), which was significantly better than that of T-SPOT.TB (AUROC = 0.692, 95%CI 0.609–0.775, *p* < 0.001) (**Figure 4B**). The logistic regression results are shown in **Supplementary Table S1**.

When the cut-off value was 0.413, the sensitivity and specificity of the triple-color FluoroSpot (IFN-γ/IL-2/TNF-α) assay differentiating ATB from LTBI were 77.1% (95%CI: 0.666–0.856) and 85.7% (95%CI: 0.764–0.924), respectively. When 302 SFCs/10^6^ PBMCs was taken as the cutoff value, the sensitivity and specificity of T-SPOT.TB differentiating ATB from LTBI were 60.2% (95%CI: 0.489–0.708) and 78.6% (95%CI: 0.683–0.868), respectively (**Table 3**). Furthermore, the difference in sensitivity between the two assays was statistically significant (*P* = 0.003), and the difference in specificity was not significant (*P* = 0.238).

## 4 Discussion

Etiological evidence is the gold standard for ATB diagnosis. However, acid-fast bacilli staining, MTB culture, Xpert *MTB*/RIF, and other etiological diagnostic methods still suffer many problems, such as low sensitivity, long time consumption, and difficulty in collecting qualified samples. The results of TST are affected by BCG vaccination and non-tuberculous mycobacteria, and the specificity is poor (21, 22). Although the specificity of IGRA is better than TST, it still cannot distinguish ATB from LTBI (23). Our previous research showed that less than 30% of patients with ATB were diagnosed by etiology in general hospitals, and the proportion of LTBI in patients who need to be differentiated from ATB was as high as 26% (24). Fever is a common clinical manifestation of many diseases, including ATB. A test that could differentiate ATB patients from other febrile ones would be very helpful in clinical practice.

This study preliminarily evaluated the accuracy of MTB-specific triple-color FluoroSpot (IFN-γ/IL-2/TNF-α) in diagnosing ATB and differentiating ATB from LTBI in febrile patients. The immunological mechanism of TB is very complex. Innate immunity and adaptive immunity are crucial for controlling MTB infection, and innate immunity mainly controls early MTB infection (25–28). Various cytokines are involved in the cellular immune response caused by MTB infection, of which IL-2, IFN-γ, and TNF-α are cytokines secreted by specific T cells at different stages of differentiation (11, 29).IFN-γ inhibits the proliferation of MTB by activating macrophages to transport MTB to lysosomes (30). IL-2 can activate dysfunctional T cells caused by sustained antigen stimulation, among several other functions, such as regulating immune homeostasis and promoting the differentiation of regulatory T cells (31). TNF-α attaches to endothelin cells and improves vascular permeability, allowing circulating neutrophils and monocytes to be recruited by inflammatory cytokines into the site of infection. Additionally, TNF-α promotes programmed necrosis of infected macrophages through the production of mitochondrial reactive oxygen species and the participation of cyclophilin D, while also contributing to the regulation of tissue damage and granuloma formation. TNF-α and IFN-γ synergistically convert inactivated macrophages into M1 subtypes, among other roles, to maintain the integrity of granulomas and sustain the immune response (32, 33).

Cytokine assays hold promise for the diagnosis and differential diagnosis of ATB and LTBI. A systematic review included 56 articles using MTB-specific cytokine biomarkers to differentiate ATB and LTBI (11). In this review, of 50 studies that measured IFN-γ, seven studies found secreting MTB-specific IFN-γ is higher in ATB than in LTBI, ten studies yielded ATB patients secreting MTB-specific IFN-γ lower than LTBI, and the remaining 33 studies found no difference between the two. The IL-2 was detected in 36 studies, of which 13 obtained differential results (The secretion of IL-2 in ATB group was higher than that in LTBI group in 4 studies and lower than that in LTBI group in 9 studies). The secretion of TNF-α stimulated by MTB in ATB and LTBI were reported in 34 articles, 9 of which obtained differential results (8 ATB was higher than LTBI, and 1 ATB was lower than LTBI). Sun, Q’s research showed that compared with IFN-γ or IL-2 alone, the use of the IL-2/IFN-γ ratio could significantly improve the sensitivity and specificity in differentiating ATB from LTBI, and the IL-2/IFN-γ ratio measured under PPD stimulation had a sensitivity and specificity of 90.8% and 97.7% in distinguishing ATB and LTBI. (34). An exploratory study in children conducted by Gourgouillon found that no significant differences in IFN-γ, IL-2, and TNF-α secreted by ATB and LTBI patients after MTB antigen stimulation, but the sensitivity and specificity of distinguishing ATB and LTBI were 83% and 87% with the TNF-α/IL-2 ratio 0.73 as the diagnostic cut-off value (35). These findings suggest that diagnosing TB infection status based on a single cytokine may be less reliable and unstable. In addition to measuring the absolute amounts of IFN-γ, IL-2, and TNF-α produced after stimulation, the relative increases in the secretion of these three cytokines may also serve as important indicators of tuberculosis infection status. The triple-color FluoroSpot assay, which eliminates spot color mixing when detecting multiple factors, enables the simultaneous detection of IFN-γ, IL-2, and TNF-α secretion by individual cells. So that we can not only know the frequencies of T cells secreting different cytokine profiles but also get the proportions of T cells of each type to assist in the diagnosis of TB infection status. Furthermore, meta-analysis has indicated that, in addition to the three cytokines examined in this study, IL-7, CCL4, and CXCL10 also show significant differences between ATB and LTBI (11). Additionally, compared to ATB, latent tuberculosis-related antigen stimulation can induce a more pronounced T cell immune response in LTBI. The combination of specific latent tuberculosis-related antigens with cytokines may help distinguish ATB from LTBI (12, 36).

In this study, triple-color FluoroSpot (IFN-γ/IL-2/TNF-α) and enzyme-linked immunospot (EliSpot) Assay simultaneously determined the total number of IFN-γ-secreting T cells stimulated by MTB-specific antigen (ESAT-6/CFP-10) in all patients. Similar to Chesov’s study (37), the SFC counts obtained by triple-color FluoroSpot (IFN-γ/IL-2/TNF-α) were lower than those obtained by EliSpot. However, there was a strong and significant correlation between IFN-γ secretion, which was similar to previous studies (15, 38). Therefore, our study further verified that triple-color FluoroSpot (IFN-γ/IL-2/TNF-α) had similar results to ELiSpot in detecting IFN-γ-secreting T cells, regardless of the type of antigen used. We found that the frequencies of IFN-γ-, IL-2-, and TNF-α-secreting T cells in MTB-infected patients (ATB and LTBI) were higher than that of uninfected patients, among which the frequencies of IFN-γ- and TNF-α-secreting T cells in ATB patients were significantly higher than that of LTBI, while there was no significant difference in the frequency of IL-2-secreting T cells. Acharya obtained similar results in whole blood (39). Currently, there is no report on the use of the combination of IFN-γ, IL-2, and TNF-α in the differential diagnosis of TB infection status. Our previous study of IFN-γ/IL-2 FluoroSpot assay (L. 13) showed that the frequencies and proportions of IFN-γ^+^IL-2^-^ T cells were significantly higher in the ATB group than in the non-ATB group. Although the frequencies of IFN-γ^-^IL-2^+^- and IFN-γ^+^IL-2^+^-secreting T cells in ATB patients were higher than that in non-ATB patients, the proportions decreased. A study of IFN-γ/TNF-α FluoroSpot assay found that the frequencies of IFN-γ^+^TNF-α^-^, IFN-γ^-^TNF-α^+^, and IFN-γ^+^TNF-α^+^ T cells in the ATB group were higher than that of LTBI (15). Several studies have shown that the IL-2/IFN-γ ratio in ATB patients is lower than that in LTBI patients (34, 40, 41). However, Gourgouillon found that the TNF-α/IL-2 ratio was higher in ATB than in LTBI (35). Based on the findings mentioned above, we hypothesized that the frequencies of specific T cells secreting IFN-γ, IL-2, and TNF-α increased after stimulation with MTB-specific antigen in patients with TB infection. Furthermore, the proportions of IFN-γ and TNF-α secreting T cells were relatively increased, and the proportion of IL-2 was relatively decreased in patients with ATB.

We conducted binary logistic regression analysis with the frequencies and proportions of specific T cells secreting different cytokine profiles after stimulation with MTB-specific antigens as independent variables and used the forward LR method to screen variables, which not only ensured the maximum use of the information obtained from the study but also selected important indicators to explain the immune responses in different TB infection status. The frequencies of IFN-γ^+^IL-2^-^TNF-α^+^-, IFN-γ^+^IL-2^-^TNF-α^–^, and IFN-γ^-^IL-2^+^TNF-α^–^specific T cell and the proportions of IFN-γ^+^IL-2^+^TNF-α^–^specific T cells were included in the logistic regression equations for diagnosing ATB and differentiating ATB from LTBI. The coefficients of frequencies of IFN-γ^+^IL-2^-^TNF-α^–^specific T cell in both equations were positive, indicating that the higher the frequencies of T cell in this subset, the higher the probability of ATB. The coefficients of the frequencies of IFN-γ^+^IL-2^-^TNF-α^+^- and IFN-γ^-^IL-2^+^TNF-α^–^specific T cells and the proportions of IFN-γ^+^IL-2^+^TNF-α^–^specific T cells were estimated to be negative, which suggested that the higher the frequencies or proportions of T cell in these subsets, the lower the probability of ATB. Many previous studies showed that, compared with ATB, the frequencies of the single IL-2-secreting, dual IFN-γ/IL-2-secreting, and the dual IFN-γ/TNF-α-secreting T cells increased in patients with LTBI and cured ATB (38, 42–44), and our study further confirmed this.

We established that an MTB-specific triple color FluoroSpot (IFN-γ/IL-2/TNF-α) assay can simultaneously detect three cytokines closely related to TB infection status and found that its diagnostic accuracy was better than that of T-SPOT.TB, which may play an important role in assisting the diagnosis of ATB, especially in differentiating ATB from LTBI. However, there were still some limitations in this study, namely: (1) This is a cross-sectional study, but an additional 30 ATB patients confirmed by etiology were added to the ATB group, which may result in selection bias of the study subjects and thus overestimate the diagnostic accuracy. In this situation, we enrolled febrile patients who required differential diagnosis from ATB to evaluate its diagnostic accuracy in clinical practice as objectively as possible; (2) This study uses T-SPOT.TB for assessing tuberculosis infection status, which may miss some MTB-infected patients who are T-SPOT.TB negative. However, there is currently no gold standard for MTB infection assessment, and T-SPOT.TB remains one of the relatively effective diagnostic methods; (3) The triple-color FluoroSpot assay has higher requirements for experimental conditions and personnel in terms of experimental operation and result interpretation, which may not be conducive for popularization in primary hospitals; and (4) The assay is an immunological diagnostic testing and influenced by the immune status of the patient. For patients receiving corticosteroids, immunosuppressants, or biologics, further evaluation is needed to assess its diagnostic performance.

## 5 Conclusion

This study established the MTB-specific triple-color FluoroSpot (IFN-γ/IL-2/TNF-α) method and preliminarily evaluated the accuracy in the differential diagnosis of TB infection status in febrile patients. This study is enlightening for the diagnosis of ATB without etiological evidence, which is a challenge faced by many doctors in general tertiary hospitals in China. Further prospective cohort studies are warranted to validate the findings.

## Data Availability

The raw data supporting the conclusions of this article will be made available by the authors, without undue reservation.
