# Supplementary material for: Diagnostic accuracy of Mycobacterium tuberculosis-specific triple-color FluoroSpot assay in differentiating tuberculosis infection status in febrile patients with suspected tuberculosis
Source: Front Immunol. 2025 Jan 8;15:1462222. doi: 10.3389/fimmu.2024.1462222 (PMC11751065; doi:10.3389/fimmu.2024.1462222)
Supplement: Supplementary file 1 [file DataSheet1.docx]

Supplementary Material

# Supplementary Data

None

# Supplementary Figures and Tables

## Supplementary Figures


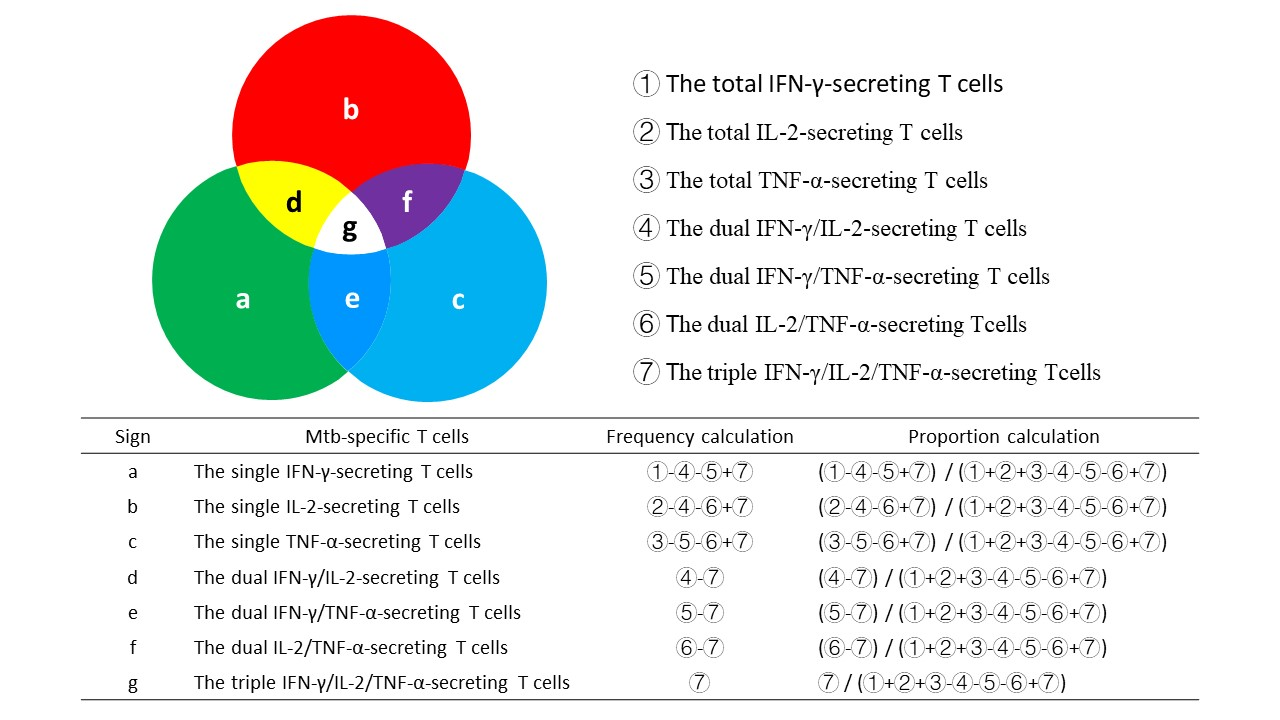


**Supplementary Figure 1.** Schematic diagram of the frequencies and proportions of cytokine-secreting specific T cells detected by MTB-specific triple-color FluoroSpot (IFN-γ/IL-2/TNF-α) assay

# Supplementary Tables

Supplement table 1. Results of logistic regression

|  | B | SE. | Wald | df | Sig. | Exp (B) | 95%CI for Exp(B) | |
| --- | --- | --- | --- | --- | --- | --- | --- | --- |
|  |  |  |  |  |  |  | Lower | Upper |
| **Distinguishing ATB from non-ATB** | | | | | | | | |
| IFN-γ^+^ IL-2^-^TNF-α^+^ | -0.225 | 0.087 | 6.691 | 1 | 0.010 | 0.798 | 0.673 | 0.947 |
| IFN-γ^+^ IL-2^-^TNF-α^-^ | 0.085 | 0.014 | 37.092 | 1 | < 0.001 | 1.088 | 1.059 | 1.118 |
| IFN-γ^-^IL-2^+^TNF-α^-^ | -0.067 | 0.023 | 8.364 | 1 | 0.004 | 0.935 | 0.893 | 0.978 |
| p_IFN-γ^+^IL-2^+^TNF-α^-^ | -4.975 | 2.358 | 4.451 | 1 | 0.035 | 0.007 | < 0.001 | 0.702 |
| Constant | -1.766 | 0.253 | 48.625 | 1 | < 0.001 | 0.171 |  |  |
|  |  |  |  |  |  |  |  |  |
| **Distinguishing ATB from LTBI** | | | | | | | | |
| IFN-γ^+^ IL-2^-^TNF-α^+^ | -0.167 | 0.078 | 4.621 | 1 | 0.032 | .846 | 0.726 | 0.985 |
| IFN-γ^+^ IL-2^-^TNF-α^-^ | 0.066 | 0.013 | 27.176 | 1 | < 0.001 | 1.068 | 1.042 | 1.095 |
| IFN-γ^-^IL-2^+^TNF-α^-^ | -0.057 | 0.020 | 7.978 | 1 | 0.005 | .945 | 0.908 | 0.983 |
| p_IFN-γ^+^IL-2^+^TNF-α^-^ | -7.106 | 2.389 | 8.849 | 1 | 0.003 | .001 | < 0.001 | 0.089 |
| Constant | -.794 | 0.287 | 7.659 | 1 | 0.006 | .452 |  |  |

p_IFN-γ^+^IL-2^+^TNF-α^-^: proportions of IFN-γ^+^IL-2^+^TNF-α^-^
